# Smoking and secondary ACL rupture are detrimental to knee health post ACL injury—a Bayesian analysis

**DOI:** 10.1186/s40634-023-00638-4

**Published:** 2023-08-09

**Authors:** Micah Nicholls, Thorvaldur Ingvarsson, Stephanie Filbay, Stefan Lohmander, Kristin Briem

**Affiliations:** 1 Faculty of Medicine, Research Centre for Movement Sciences, The University of Iceland, Sæmundargata 2, Reykjavík, 102 Iceland; 2 Department of Orthopaedic Surgery, University of Akureyri, Akureyri, Iceland; 3 Department of Physiotherapy, Faculty of Medicine Dentistry and Health Sciences, The University of Melbourne, Melbourne, Australia; 4 Department of Clinical Sciences Lund, Orthopaedics, Lunds Universitet, Lund, Sweden

**Keywords:** Knee injury, Patient-reported outcomes, ACL reconstruction, Nonoperative management, National cohort, Bayesian

## Abstract

**Purpose:**

To identify potential prognostic factors for patient-reported outcomes in an Icelandic cohort of ACL injured subjects.

**Methods:**

All knee MRI reports written in Iceland between the years 2001 to 2011 were read to identify individuals with a possible ACL injury. These individuals were contacted and asked to complete an online questionnaire regarding their injury and current knee related health. The questionnaire collected information on years since surgery, injury circumstance, brace use, physiotherapy, ACL surgery, second ACL injury and current smoking status. In addition, the baseline status of their meniscii were assessed from the original MRI report and medical records were used to identify any subsequent, non-ACL surgery. The patient-reported Knee Osteoarthritis and Injury Outcome Score (KOOS) was used assess current knee related health. A Bayesian proportional odds model was used to assess the effect of all potential prognostic factors above as well as age and sex on KOOS outcomes.

**Results:**

A total of 408 subjects completed the questionnaire indicating that they did rupture their ACL. The following variables were associated with worse outcomes across all KOOS subscales: having a subsequent arthroscopy, reinjury to your ACL, and smoking. Having physiotherapy for 9 months was associated with worse KOOS pain scores than having 6 months of physiotherapy. Conversely KOOS pain score tended to be higher if you injured your knee during sports.

**Conclusion:**

Reinjuring your ACL, smoking and having subsequent (non-ACLR) surgery predict your knee related health following an ACL injury. Strategies should be implemented to reduce the risk of secondary ACL injury, and patients should be strongly advised not to smoke.

## Introduction

The incidence of anterior cruciate ligament (ACL) rupture is 75 per 100 000 [29], affecting over 220 000 people each year in the USA alone. In the short-term, knee function and stability are compromised; in the long-term knee related function and symptoms deteriorate [33] and there is a risk of knee osteoarthritis (OA) with a recent umbrella meta-analysis reporting an almost sevenfold increase in the odds of knee OA irrespective of treatment or concomitant injury [42]. Treatment should thus focus on restoring function in the short term and minimizing the deterioration in knee health and osteoarthritis risk in the long-term. Current treatment options are either rehabilitation alone with optional delayed surgery, or surgery plus rehabilitation, with high quality randomized control trials (RCT) supporting no difference in mean outcomes between the two approaches at two to five years post injury [16, 17, 32].

To properly advise and treat patients with ACL injury it is important to understand which variables can influence both the long and short-term course of this problem. However, attempts at investigating the success of ACL treatment are hampered by ethical and practical issues. Dropouts from an interventional study are likely to be high and we can’t expect patients randomly assigned to rehabilitation to stay in that treatment group. A high-quality RCT found 51% of the group initially assigned to rehabilitation crossed over to surgery within five years [17]. Furthermore, surgical techniques change over time and most systematic reviews with long term follow up include studies using open ACL repair [21, 25, 30], a technique that is not in common practice today.

In addition to treatment, many factors, both psychological and functional, have been associated with outcomes [14]. Some variables, such as meniscal injury, do not lend themselves to randomization and so observational studies are the study design of choice. Furthermore, large numbers of subjects are typically necessary to control for multivariable analyses and this is more easily achieved using a non-interventional design. Previous multivariable analyses of ACL injured cohorts have mainly focused on surgically treated cohorts [10, 24, 31, 37, 38]. One previous multivariable analysis included both surgically and non-surgically treated patients between 15 and 40, but the surgical technique was an ACL repair rather than the more commonly used ACL reconstruction [13]; another was limited to an exploratory analysis of previously published data [15] of individuals between 15 and 35. No previous studies have investigated these factors in a sizeable national cohort of all ages including both surgically and non-surgically treated patients. Iceland offers a unique opportunity to recruit a large sample of the population because of its relatively small population and the ease of connecting medical records.

The aim of our exploratory study was to identify potential prognostic factors for patient-reported outcomes following ACL injury in an Icelandic cohort.

## Methods

### Participants and design

This study was approved by the National Bioethics Committee of Iceland (Vísindasiðanefnd, VSNb2011100031/03.07). All knee MRI examination reports taken in Iceland during the period 2001–2011 were collected and entered into a spreadsheet. These were collected from the databases of the four clinics in Iceland that can take knee MRIs. A software program was written to identify phrases relating to the status of the ACL ligament. Based on the language in the reports the ACL was thus classified as being either torn, reconstructed, normal or unclear (ambiguous report and/or low quality of the image). Reports that the software was unable to classify were read individually and put in the same categories. This methodology and its validity was previously described [29]. Letters were sent to all individuals whose report was classified as torn, reconstructed, or unclear inviting them to participate in an online questionnaire. Informed consent was obtained online prior to completing the questionnaire.

Subjects were asked to complete the Knee Osteoarthritis and Injury Outcome Score (KOOS). The KOOS is a common patient reported outcome measure that can be used in both the immediate, acute aftermath of knee injury and in the later stages of chronic pain and osteoarthritis [34]. It consists of 5 subscales: pain, symptoms, activities of daily living (ADL), sports and recreational function (Sport/Rec) and knee-related quality of life (QOL), each scored separately ranging from 0 (worst possible score) to 100 (best possible score). The KOOS subscales have been shown to have good internal consistency, test–retest reliability and construct validity in various populations including those with knee OA and ACL injury [6]. The KOOS has been translated and validated in Icelandic [4]. In addition to the KOOS, respondents answered questions about the timing and circumstances of their injury as well as the treatment. An individual’s responses were included in the analysis if they responded “yes” to the question “Have you ever torn your anterior cruciate ligament?”.

### Prognostic and confounding factors

Knowledge of the literature and clinical reasoning were used to initially select potential prognostic and confounding variables. These variables were entered into a directed acyclic graph (DAG) [40] which helped to identify possible colliders [5] and avoid over adjustment bias [35]. The following variables were included in the model: age at injury, years since injury and physiotherapy (number of months), sex, ACLR (yes/no), subsequent arthroscopic knee surgery (not revision ACLR, yes/no), meniscal injury (yes/no); subsequent ACL injury (ipsilateral, contralateral or both knees), current smoker (daily, less than daily or none), brace use (yes/no). All data other than subsequent arthroscopic surgery and meniscal injury were collected from the questionnaire. Subsequent arthroscopic surgery was recorded by linking the subjects’ national ID number with national insurance records, which capture all such procedures performed in Iceland. Several codes within the national insurance records are used to bill for knee arthroscopic surgery, however they do not clearly indicate the type of surgery performed. Meniscal injuries were identified from the initial MRI report.

### Statistical analysis

All data analyses were performed using R 4.0.3 (Vienna, Austria). A Bayesian multivariable proportional odds model was used to quantify the odds ratio (OR) between levels of the KOOS subscale and levels of the factors listed above. The model was implemented using the blrm function from the rmsb package. Missing data were imputed using the aregImput function from the Hmisc package in R. stackMI from the rmsb package was used to run the analyses on each imputed dataset, amalgamate the results and compute parameter summaries [20]. Non-informative priors were modelled using the Dirichlet distribution. An adjusted OR greater than 1.0 indicates a more favourable outcome on the KOOS subscale. Number of months of physiotherapy, age at injury and years since injury were modelled using a restricted cubic spline to allow for a nonlinear relationship. We selected the maximum number of variables our model could accommodate to be the effective sample size divided by 20 [20]. When reporting the OR for continuous variables we chose to report the OR for the interquartile range which is where the bulk of the data lie and thus offers the most robust comparison. For months of physiotherapy, we report the OR of 9 months vs 6 months, that is the odds of having a higher vs a lower score at 9 months divided by the odds of having a higher vs a lower score at 6 months. This contrasts commonly held beliefs amongst physiotherapists on the necessary rehabilitation period prior to return to sports [11, 12, 18].

Bayesian inference allows for a straightforward interpretation of the existence of the alternative hypothesis. For example, we can directly state the probability of OR associated with a parameter being greater than one. If further allows us incremental knowledge gain.

Variables were determined to be significant if the 95% highest posterior density interval (HPDI) of their model coefficient did not include 1. For those variables the contrast function in the rms package was used to calculate the actual effect on KOOS scores to ease interpretation. Plots of these effects included the minimum detectable change (MDC) as described by Collins et al. [6]. The minimum clinical important difference (MCID) was not included for several reasons. The value of the MCID can change dramatically based on many factors including the study population, the trial design, the disease severity as well as the methods used to derive its value [28]. As such there is unlikely to be one valid MCID that can be applied to this study. In addition, the outcomes presented were taken for each individual at a single point in time and thus the MCID cannot be used to assess whether individuals improved by a clinically meaningful amount.

Differences between questionnaire responders and non-responders were tested using a Wilcoxon test for continuous variables and Pearsons test for binary variables.

## Results

### Participant characteristics

**Fig. 1:**
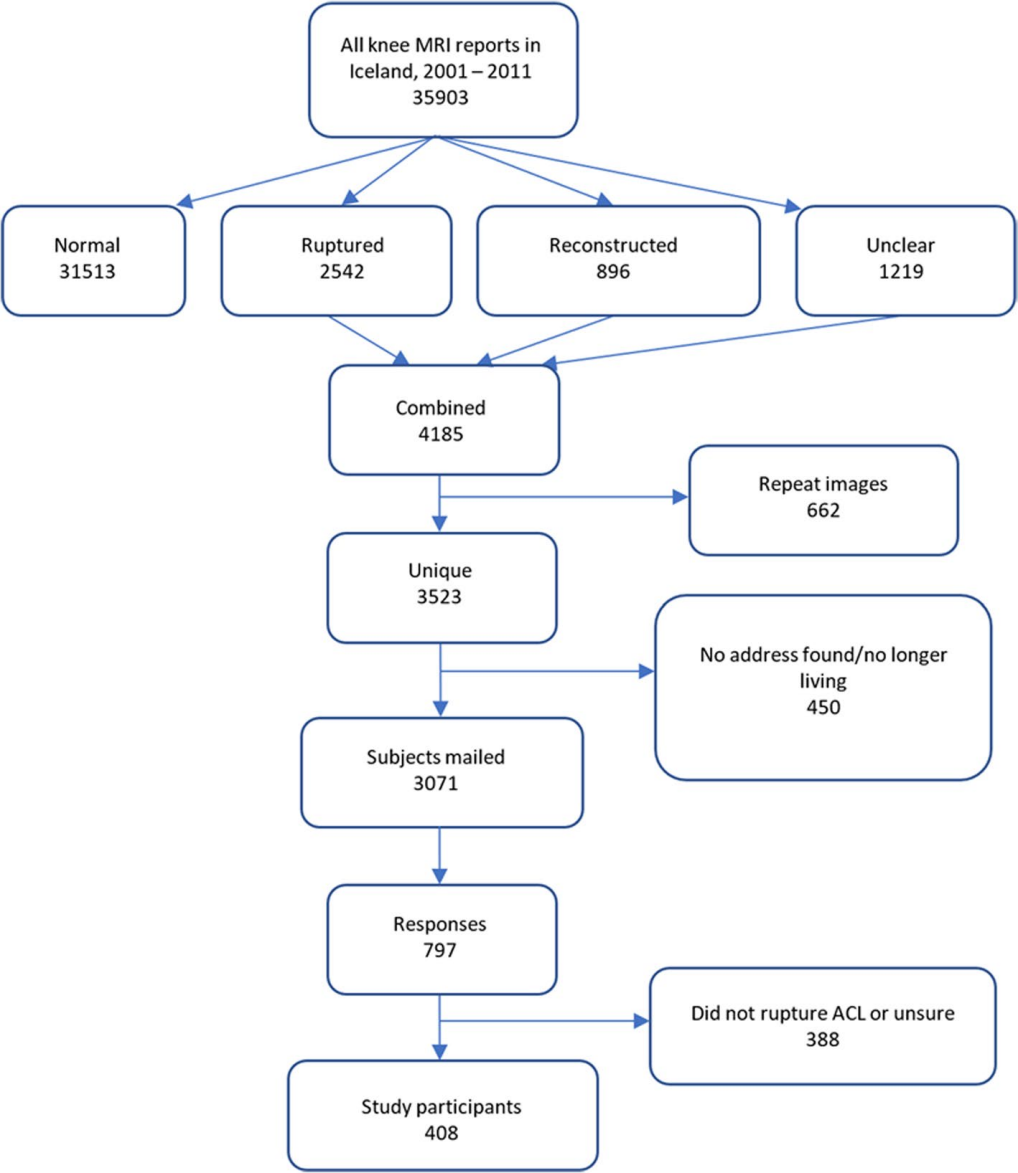
Classification of magnetic resonance imaging (MRI) reports and development of cohort

A total of 35,903 knee MRIs were performed in Iceland between 2001 and 2011. Of the associated reports 31,513 were determined to have no injury to the ACL. After accounting for duplicates 3,523 remained consisting of ACL ruptures, ACL reconstructions and reports where the ACL status was unclear. Invitation letters were mailed to 3071 for whom valid addresses could be found and 797 responses were received. Of those, 408 indicated that they had torn their ACL and were used for this analysis, Fig. 1. The majority of ACL injured respondents, 323 (79%), had an ACLR. The average age of eligible participants was 39 years (IQR 31–50 years) and the number of years since the ACL rupture occurred ranged between 3 and 40 years (median 11 years, IQR 7–13 years). Table 1 describes the cohort in more detail. 96% (*n* = 312) of participants who stated that they had an ACLR were operated during or after the year 2000.

**Table 1:** Cohort demographics

	N	Male	Female	Combined
		*N = 234*	*N = 174*	*N = 408*
Age at injury	408	22.0 **29.0** 36.0	17.0 **25.0** 39.8	20.0 **28.0** 37.0
		29.9 ± 10.1	29.2 ± 13.7	29.6 ± 11.7
Years since injury	408	7.00 **10.00** 14.00	7.00 **9.00** 12.75	7.00 **10.00** 13.00
		11.25 ± 5.91	10.82 ± 6.47	11.07 ± 6.15
Injury during sports	408	0.88 (206)	0.79 (137)	0.84 (343)
ACLR	408	0.82 (192)	0.75 (131)	0.79 (323)
Physiotherapy duration (months)	399	2.00 **4.00** 7.00	3.00 **6.00** 10.00	2.00 **4.00** 7.00
		4.53 ± 3.62	6.32 ± 4.30	5.28 ± 4.01
Used a brace	408	0.52 (122)	0.53 (92)	0.52 (214)
Current smoker: never	408	0.88 (205)	0.85 (148)	0.87 (353)
daily		0.03 (8)	0.08 (14)	0.05 (22)
less than daily		0.09 (21)	0.07 (12)	0.08 (33)
Second ACL injury: none	408	0.66 (154)	0.71 (123)	0.68 (277)
ipsilateral		0.13 (30)	0.16 (28)	0.14 (58)
contralateral		0.12 (28)	0.09 (15)	0.11 (43)
both		0.09 (22)	0.05 (8)	0.07 (30)
Subsequent arthroscopy	308	0.33 (55)	0.42 (58)	0.37 (113)
Meniscus injury	408	0.78 (183)	0.74 (129)	0.76 (312)
KOOS pain	408	77.8 **88.9** 97.2	72.2 **86.1** 94.4	75.0 **88.9** 97.2
		85.5 ± 15.1	80.9 ± 18.7	83.5 ± 16.8
KOOS symptoms	408	64.3 **78.6** 89.3	57.1 **78.6** 89.3	64.3 **78.6** 89.3
		76.4 ± 18.3	72.0 ± 22.5	74.5 ± 20.3
KOOS activities of daily living	408	86.8 **98.5** 100.0	80.9 **95.6** 100.0	85.3 **97.1** 100.0
		91.7 ± 11.8	87.4 ± 17.2	89.9 ± 14.5
KOOS sports and recreation	408	50.0 **75.0** 90.0	35.0 **65.0** 85.0	45.0 **75.0** 90.0
		67.1 ± 27.5	60.9 ± 30.2	64.5 ± 28.8
KOOS quality of life	408	50.0 **68.8** 81.2	37.5 **62.5** 81.2	43.8 **68.8** 81.2
		64.8 ± 24.1	60.3 ± 26.3	62.9 ± 25.1

a **b** c represents the lower quartile a, the median b, and the upper quartile c for continuous variables. x ± s represents the mean ± 1 standard deviation. Numbers after proportions are frequencies

*ACLR* Anterior Cruciate Ligament Reconstruction, *ACL* Anterior Cruciate Ligament, *KOOS* Knee injury and Osteoarthritis Outcomes Score

There were differences in age at the time of injury and sex between study participants and those who were identified as having a potential ACL rupture (Table 2). Usable responses came from people who tended to be younger (29 vs 35, *p* < 0.001), and more likely to be female (63% vs 44%, *p* < 0.001).

**Table 2:** A comparison of all subjects with a potential ACL rupture to those mailed subjects who were included in the analysis

	Identified as having an ACL tear	Study participant	Test statistic
	*N = 3115*	*N = 408*	
Age at injury	24.0 **35.0** 48.0	21.0 **29.5** 41.0	*F*_1 3521 _= 36.1, *P *< 0.001^1^
	37.0 ± 15.5	31.9 ± 12.4	
Sex: female	0.34 (1051)	0.43 (174)	${}_1^2=12.6$, *P *< 0.001^2^

a **b** c represents the lower quartile a, the median b, and the upper quartile c for continuous variables. x ± s represents the mean ± 1 standard deviation. Numbers after proportions are frequencies. Tests used: ^1^Wilcoxon; ^2^Pearson test

### Prognostic factors for patient-reported outcomes following ACL injury

**Fig. 2:**
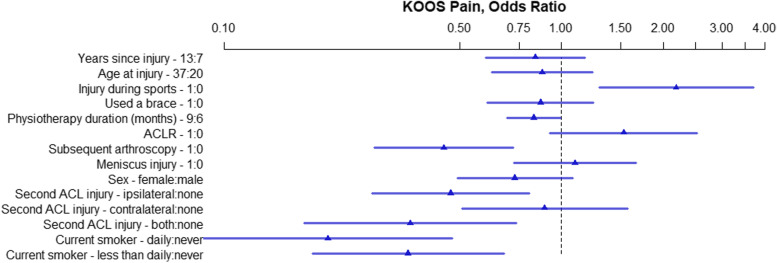
KOOS Pain, OR of model parameters. The numbers following the variable name indicate the variable levels used for the OR. For binary variables 1 = yes, 0 = no. For continuous variables the interquartile range in years is used. For physiotherapy 9 months and 6 months. ACL – Anterior Cruciate Ligament; ACLR – Anterior Cruciate Ligament Reconstruction; KOOS – Knee injury and Osteoarthritis Outcomes Score; OR – Odds Ratio

**Fig. 3:**
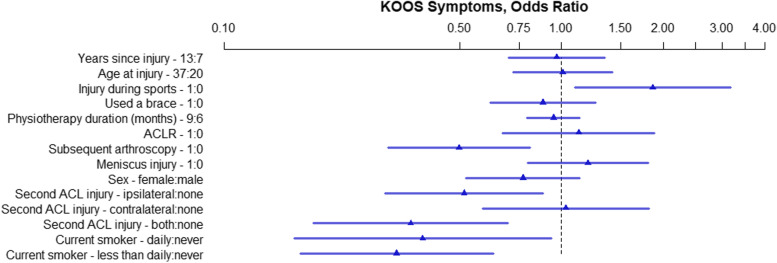
KOOS Symptoms, OR of model parameters. The numbers following the variable name indicate the variable levels used for the OR. For binary variables 1 = yes, 0 = no. For continuous variables the interquartile range in years is used. For physiotherapy 9 months and 6 months. ACL – Anterior Cruciate Ligament; ACLR – Anterior Cruciate Ligament Reconstruction; KOOS – Knee injury and Osteoarthritis Outcomes Score; OR – Odds Ratio

**Fig. 4:**
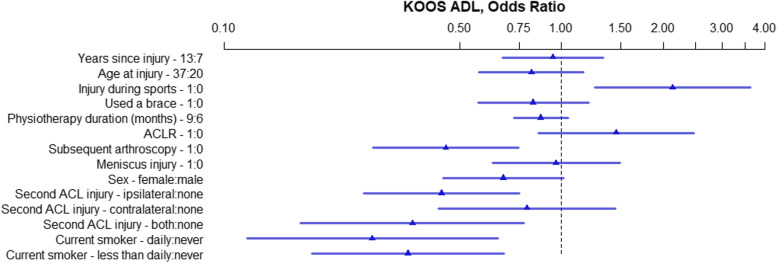
KOOS ADL, OR of model parameters. The numbers following the variable name indicate the variable levels used for the OR. For binary variables 1 = yes, 0 = no. For continuous variables the interquartile range in years is used. For physiotherapy 9 months and 6 months. ACL – Anterior Cruciate Ligament; ACLR – Anterior Cruciate Ligament Reconstruction; KOOS – Knee injury and Osteoarthritis Outcomes Score; OR – Odds Ratio; ADL – Activities of Daily Living

**Fig. 5:**
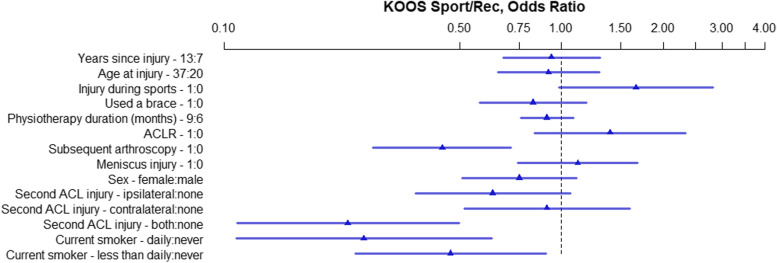
KOOS Sport/Rec, OR of model parameters. The numbers following the variable name indicate the variable levels used for the OR. For binary variables 1 = yes, 0 = no. For continuous variables the interquartile range in years is used. For physiotherapy 9 months and 6 months. ACL – Anterior Cruciate Ligament; ACLR – Anterior Cruciate Ligament Reconstruction; KOOS – Knee injury and Osteoarthritis Outcomes Score; OR – Odds Ratio; Sport/Rec – Sports and Recreation

**Fig. 6:**
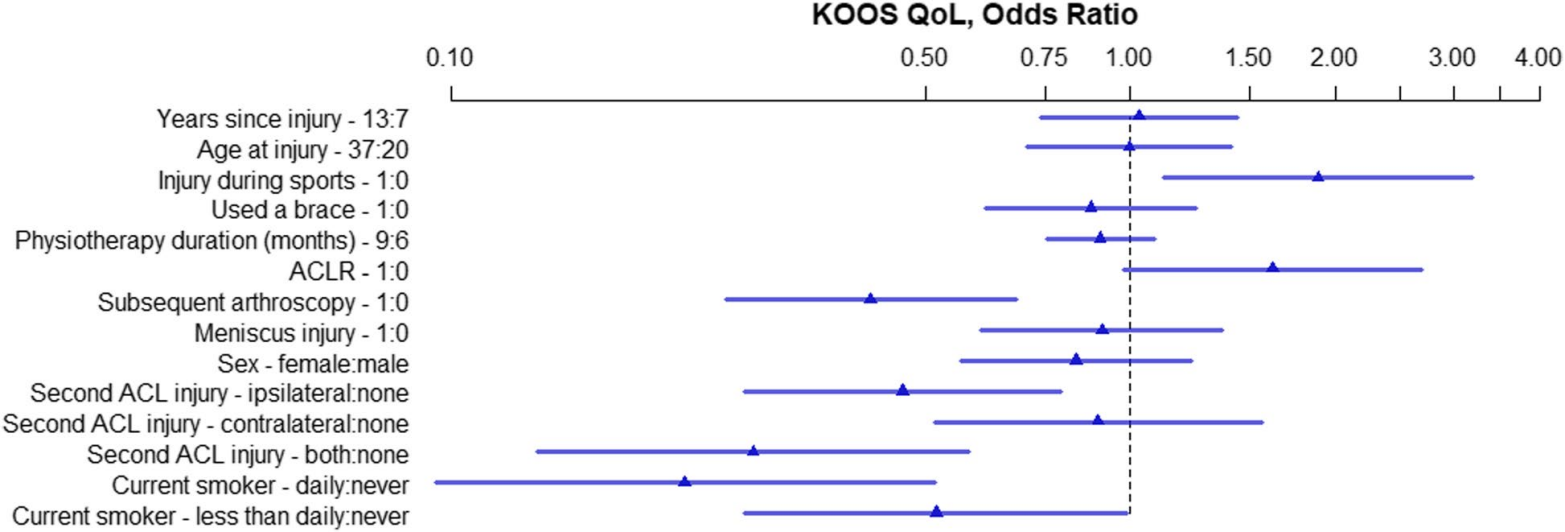
KOOS Quality of Life, OR of model parameters. The numbers following the variable name indicate the variable levels used for the OR. For binary variables 1 = yes, 0 = no. For continuous variables the interquartile range in years is used. For physiotherapy 9 months and 6 months. ACL – Anterior Cruciate Ligament; ACLR – Anterior Cruciate Ligament Reconstruction; KOOS – Knee injury and Osteoarthritis Outcomes Score; OR – Odds Ratio; QoL – Quality of Life

The OR and 95% HPDI for each variable within the five models created for the five KOOS subscales are presented in Figs. 2, 3, 4, 5, and 6. Consistently across all subscales the upper limit for the 95% HPDI of the OR was below 1 for the following variables: Subsequent arthroscopy, reinjury to the ACL of both knees, and smoking. Thus, the odds of having a higher score were consistently improved across the different KOOS subscales in those who did not have a subsequent arthroscopy, who did not reinjure their ACL and who do not smoke. Similarly, injuring your ACL during sports was related to higher KOOS subscale scores (with the lower limit of the 95% HPDI being above 1 for all subscales other than KOOS Sport/Rec. Conversely, the odds of having a higher KOOS Pain score were decreased with 9 months of physiotherapy in comparison to 6 months, Fig. 2.

The following factors did not exclude an OR of 1 on any of the KOOS subscales meaning they are unlikely to be associated with the KOOS outcome: Years since injury, age at injury, use of a brace, meniscus injury and sex.

**Fig. 7:**
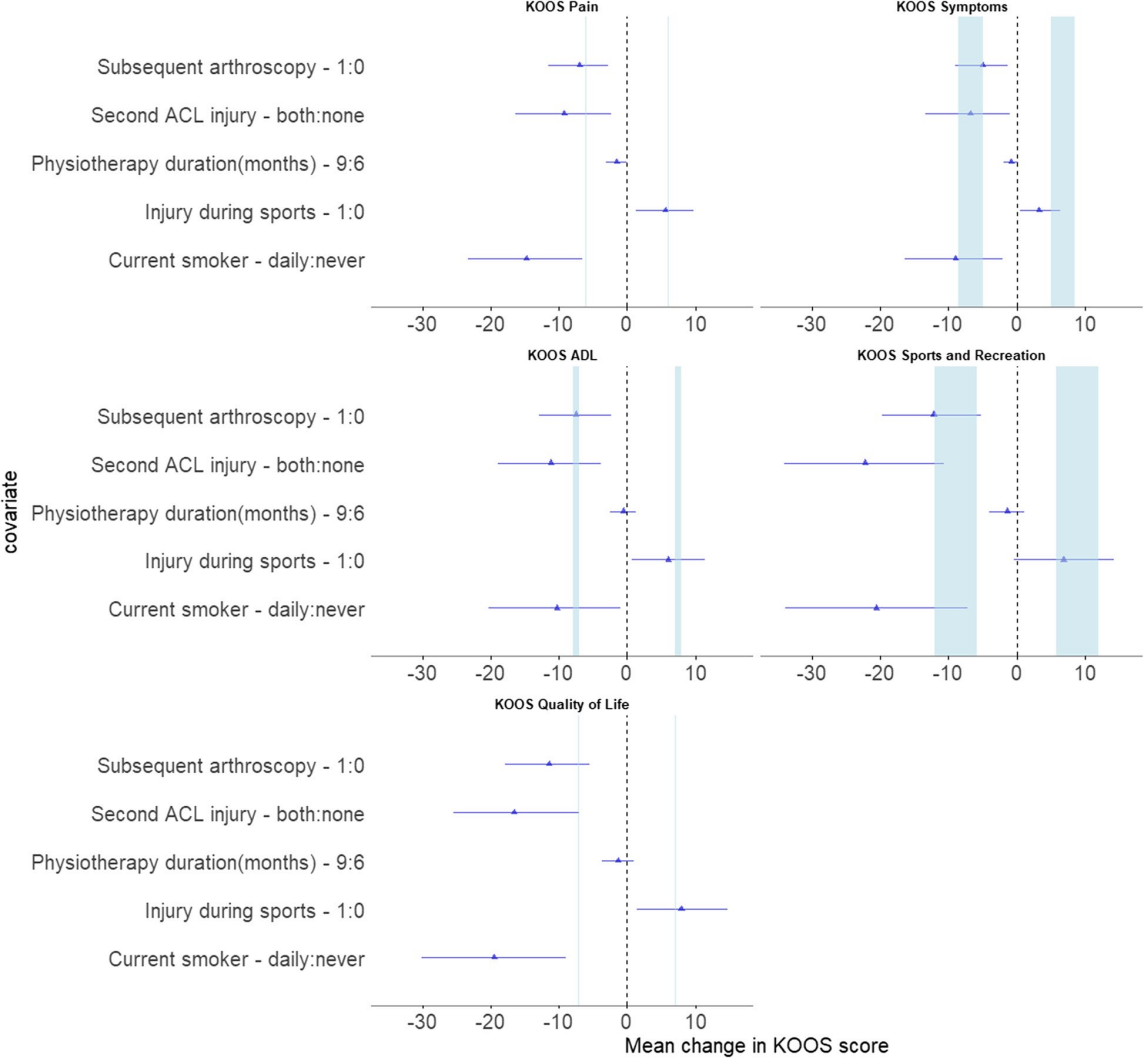
Effect of variables whose HPDI did not cross 1 on KOOS score, mean ± 95% HPDI. Blue bars represent range of minimal detectable change [6]. ACL – Anterior Cruciate Ligament; ACLR – Anterior Cruciate Ligament Reconstruction; KOOS – Knee injury and Osteoarthritis Outcomes Score; ADL – Activities of Daily Living

Figure 7, shows the effect on KOOS scores of variables with an HPDI excluding 1 and MDC for each of the KOOS subscales. The values for physiotherapy fall well below published values for MDC.

## Discussion

In this cross-sectional survey of individuals who had injured their ACL between 3 and 40 years previously (IQR 7—13), and when considering the largest OR magnitude with HPDI excluding 1, the top three variables negatively related to their knee health and quality of life were smoking, reinjuring the ACL and having a subsequent arthroscopy. In contrast, having injured your ACL during sports was related to an increase in odds of a higher KOOS outcome.

Previous studies taking a multivariable approach have investigated various factors affecting knee health post ACL injury [8, 10, 13, 15, 24, 31, 37, 38]. The majority are observational studies focusing on ACLR patients [8, 10, 24, 31, 37, 38]. All but one, [10], include the KOOS as a patient reported outcome measure. In addition to multivariable regression, two studies [24, 38] used a stepwise approach to identify covariates, which causes a number of serious problems [36]. Such problems include underestimation of the standard error resulting in overly narrow confidence intervals, and a low chance of selecting the correct covariates.

Although our cohort contained both surgically and non-surgically treated individuals the results regarding smoking and secondary injury to the ipsilateral knee are consistent with surgically treated cohorts in that knee health was negatively associated with smoking [10, 37] and secondary injury to the ipsilateral knee [31]. This is unsurprising as the inclusion of non-surgically repaired subjects in our study is unlikely to result in a different effect of these variables. Smoking is well-known to impair tissue repair via vasoconstriction and its reduction of collagen synthesis [23, 43]. In addition there may also be an inverse relationship between smoking and healthy behaviours such as exercise [7]. Smoking accounted for 13% of the cohort, secondary injury for 32% and both these variables are amenable to intervention. Thus, outcomes for a meaningful proportion of patients could potentially be improved. Indeed, training programmes have been shown to dramatically reduce secondary ACL injuries [2].

We found subsequent arthroscopy to be negatively related to knee health, however it is not possible to determine the exact nature of those operations. Within the Icelandic national insurance system there are several descriptors used to indicate knee arthroscopy. The descriptor “Arthroscopy with shaver” accounted for 81% of the subsequent arthroscopies in our cohort and a further 17% used “Arthroscopy with operation on meniscus/plica/corpus liberum” as their descriptor. However, as arthroscopic meniscectomy is the most common surgical procedure [9] it is likely that meniscectomy accounts for the majority of these surgeries. While this classification is particular to the Icelandic healthcare system a similar covariate described as “non-ACL surgeries” was included in an exploratory analysis of previously reported data from an RCT comparing early ACLR to rehabilitation plus optional delayed ACLR [15]. In this case one or more surgeries between baseline and 5 year follow up was also associated with worse KOOS outcomes. Surgery to the medial vs lateral meniscus may have differing effects on outcomes [3, 8, 10, 27] but we were unable to elicit these because of the nature of our data.

Previous research differs on the effect of sex on KOOS outcomes [1, 8, 19]. In a 20-year observational follow up of ACLR patients, Hagemans et al. [18] found females to have worse KOOS outcomes. In contrast Cox et al. [7] showed no difference in KOOS outcomes for an ACLR cohort followed up at 2 and 6 years. It is possible that there is an interaction between sex and time-to-follow-up such that over time, females improve at a slower rate. In this case the earlier follow up by Cox et al. and the lack of interaction terms in our model may fail to show an effect of sex. Alternatively, both our study and Cox et al. used a proportional odds model adjusted for covariates whereas Hagemans et al. used Mann Whitney test with no adjustment for covariates. Indeed, Ageberg et al. [1] showed worse KOOS scores for females at 1–2 years post ACLR but this difference dimished once they accounted for baseline KOOS scores.

While we did not show an effect of years since injury, for statistical reasons we chose to compare the upper and lower quartile ages as this is where the bulk of our data lies. It is likely that a greater gap would show a difference as KOOS measured symptoms have been shown to improve up to 1 to 2 years and then steadily deteriorate subsequently [26].

Other studies do not appear to have compared patients who injured their knee during sports with those who injured their knees in other situations. Approximately 15% (*n* = 65) of our cohort did not injure their ACL during sports and had worse outcomes on all KOOS subscales. Of those, 44 subjects entered an open text response to their activity at the time of injury, with the majority reporting a fall. Athletes’ increased tolerance to pain and their greater quality of life compared to the general population may offer some explanation [22, 39] as to why injury during sports might confer better outcomes. Alternatively, those injured during a fall may have sustained additional injuries leading to worse outcomes.

The finding that 9 months of physiotherapy reduced the odds of a favourable KOOS Pain outcome in comparison to 6 months is counterintuitive and unlikely to reflect a deleterious effect of excess physiotherapy. It is more likely that patients who were in worse condition required more physiotherapy. However, the magnitude of the OR, when translated into actual KOOS scores, appears to be far below the MDC (Fig. 7) and is therefore unlikely to be clinically meaningful.

Of those who reported having an ACLR, (*n* = 323 vs. 85 with no ACLR) most were operated on after 2000. Since 2000, all ACLR surgery in Iceland has been done arthroscopically, the vast majority using a graft harvested from the hamstrings. Unfortunately, nothing is known about their rehabilitation protocol other than the patient reported number of months of physical therapy. So, while the results failed to show a clear difference for those having an ACLR this lack of information on the rehabilitation protocols used for either group would make it difficult to interpret any such difference if it did exist. Nevertheless, to date only two high quality RCTs comparing early surgery plus rehabilitation to rehabilitation plus optional delayed surgery have been performed and neither show superiority of one arm over the other. Rather they both suggest that rehabilitation plus optional delayed surgery may improve overall outcomes by limiting surgery to those who need it [16, 17, 32]. In contrast to these outcomes, a 10 year follow up of an RCT comparing conservative treatment to surgical reconstruction of an ACL injury showed better subjective IKDC outcomes for the surgically treated group [41]. However, these results are hampered by a number of issues such as a small sample size and lack of control for important covariates.

Our study was unique in several ways. Although our response rate was low, we performed a multivariable analysis of observational data taken from a nationwide cohort without regard for age, gender, injury mechanism or any other factor. This improves the ability to project our results onto the general population of patients with ACL injury regardless of whether they were surgically treated, injured their ACL during sports, or were young at the time of injury, all common limitations of previous studies. In addition, the use of national insurance records to show subsequent arthroscopic surgery in this cohort is, to our knowledge, unique. We chose to use a Bayesian proportional odds model to analyse the data. Previous authors have either taken a non-parametric approach, which precludes the inclusion of continuous covariates, or assumed that the scale was continuous and used a multivariable linear model. While KOOS subscale outcomes are presented as continuous the questions are answered on a Likert scale with choices for each item being ordered nominal values. Thus, the assignment of numeric values to those choices are arbitrary and the scale is not truly continuous. In addition, the scores are bounded between 0 and 100. The proportional odds model makes fewer distributional assumptions than linear regression and may thus be a more appropriate model.

There are several limitations to our study. Importantly the accuracy of the patient reported data will be affected by patient recall. In some cases, the initial ACL injury occurred over 30 years previously although most responses were within 13 years. It is not possible to know the true proportion of the ACL injured population that responded or the reasons why they responded. Many letters were likely sent to people without an ACL tear as MRI reports with unclear ACL status were included. Indeed, of the returned questionnaires, 49% stated that they had not injured their ACL. It is further possible that people with worse knee health are more motivated to complete the questionnaire and those who were operated more likely to know that their ACL was torn, biasing the results towards those who are worse off or operated.

In addition, all limitations of a cross-sectional retrospective cohort apply. In particular, we cannot make causal conclusions, although given the temporal relationship between variables such as ACLR and current knee health, only one direction for causation may be possible. Lastly our inability to control for BMI and baseline knee health, both of which we identified as possible confounders, may affect the results.

## Conclusion

In conclusion, this cross-sectional study of 408 ACL injured patients 3 to 40 years post injury showed that the most important factors associated with knee health in ACL injured individuals are smoking, reinjuring your ACL and having a subsequent arthroscopic surgery. While the nature of the study precludes causal conclusions patients should be cautioned against smoking and strategies implemented to reduce the risk of secondary injury.
